# Identification of immunosuppressive signature subtypes and prognostic risk signatures in triple-negative breast cancer

**DOI:** 10.3389/fonc.2023.1108472

**Published:** 2023-06-12

**Authors:** Ran Ding, Yuhan Wang, Jinyan Fan, Ziyue Tian, Shuang Wang, Xiujuan Qin, Wei Su, Yanbo Wang

**Affiliations:** ^1^ Changchun University of Chinese Medicine, Changchun, Jilin, China; ^2^ Anhui University of Chinese Medicine, Hefei, Anhui, China; ^3^ The Affiliated Hospital of Changchun University of Chinese Medicine, Jilin, China; ^4^ Department of Traditional Chinese Medicine, The First Hospital of Jilin University, Changchun, Jilin, China

**Keywords:** immunogenomics, TNBC, t cell exhaustion, immunosuppressive cytokines, tumor microenvironment

## Abstract

**Purpose:**

Immune checkpoint blockade (ICB) therapy has transformed the treatment of triple-negative breast cancer (TNBC) in recent years. However, some TNBC patients with high programmed death-ligand 1 (PD-L1) expression levels develop immune checkpoint resistance. Hence, there is an urgent need to characterize the immunosuppressive tumor microenvironment and identify biomarkers to construct prognostic models of patient survival outcomes in order to understand biological mechanisms operating within the tumor microenvironment.

**Patients and methods:**

RNA sequence (RNA-seq) data from 303 TNBC samples were analyzed using an unsupervised cluster analysis approach to reveal distinctive cellular gene expression patterns within the TNBC tumor microenvironment (TME). A panel of T cell exhaustion signatures, immunosuppressive cell subtypes and clinical features were correlated with the immunotherapeutic response, as assessed according to gene expression patterns. The test dataset was then used to confirm the occurrence of immune depletion status and prognostic features and to formulate clinical treatment recommendations. Concurrently, a reliable risk prediction model and clinical treatment strategy were proposed based on TME immunosuppressive signature differences between TNBC patients with good versus poor survival status and other clinical prognostic factors.

**Results:**

Significantly enriched TNBC microenvironment T cell depletion signatures were detected in the analyzed RNA-seq data. A high proportion of certain immunosuppressive cell subtypes, 9 inhibitory checkpoints and enhanced anti-inflammatory cytokine expression profiles were noted in 21.4% of TNBC patients that led to the designation of this group of immunosuppressed patients as the immune depletion class (IDC). Although IDC group TNBC samples contained tumor-infiltrating lymphocytes present at high densities, IDC patient prognosis was poor. Notably, PD-L1 expression was relatively elevated in IDC patients that indicated their cancers were resistant to ICB treatment. Based on these findings, a set of gene expression signatures predicting IDC group PD-L1 resistance was identified then used to develop risk models for use in predicting clinical therapeutic outcomes.

**Conclusion:**

A novel TNBC immunosuppressive tumor microenvironment subtype associated with strong PD-L1 expression and possible resistance to ICB treatment was identified. This comprehensive gene expression pattern may provide fresh insights into drug resistance mechanisms for use in optimizing immunotherapeutic approaches for TNBC patients.

## Introduction

Triple-negative breast cancer (TNBC) is a term used to describe a subset of breast cancers (BCs) defined by their lack of expression of oestrogen receptors, progesterone receptors and human epidermal growth factor receptors (1, 2). The clinical management of TBNC, a highly heterogeneous disease, is a great challenge, due to high incidence rates of visceral TNBC metastases and a lack of recognized therapeutic targets. As compared with stage 1 TNBC patients, patients with stage II or III TNBC are at greater risk of disease recurrence and death, such that at 5 years post-diagnosis, the event-free survival rate is only about 71% and the overall survival rate is only about 77% (3).

Current strategies for predicting treatment outcomes and making treatment decisions are typically based on cancer cell histologic subtype and clinical parameters (e.g., disease stage, metastasis and tumor resectability). However, recently developed molecular profiling methodologies have enabled clinicians to quantitatively analyze tumors based on genome-wide gene transcription profiles, protein expression profiles and/or mutation profiles. In turn, use of these powerful methods has made it possible to define tumor subtypes more accurately and precisely in order to achieve improved prediction of therapeutic responses of specific tumor types to specific treatments. For example, Perou et al. utilized these methods to obtain gene expression-based cellular signatures that were used to classify BC cells into five intrinsic molecular subtypes: basal-like, normal-like, HER2-enriched, luminal A and luminal B. Notably, this classification scheme aligned with tumor subtype differences related to tumor cell origin and differential progression characteristics. Meanwhile, results obtained by another group identified three BC subtypes (1q/16q, amplifier and complex) based on gene copy number alteration (I) patterns (4).

It is well known that cancer initiation, progression and therapeutic resistance are influenced by genetic and epigenetic changes that, in turn, are influenced by crosstalk between tumor cells and the local tumor microenvironment (TME). In fact, TME immune cell infiltration is associated with improved BC patient clinical outcomes when infiltration occurs at high levels and thus can serve as a valuable prognostic marker (5, 6). In particular, higher CD8+ T cell infiltration levels are strongly associated with better overall survival (OS) in oestrogen receptor (ER)-negative BC patients (7, 8), while high-level immune cell infiltration has been associated with enhanced responses to adjuvant chemotherapy (9). In recent years, numerous studies have shown that transcriptome data can be useful for describing the TME (10–15). For example, results of several studies suggest that high TME expression of leukocyte-related genes is associated with lower BC recurrence risk (10, 13, 16, 17). Notably, results of recent meta-studies reported by Ali et al. and Bense et al. have provided insights into how specific immune cell types within the TME affect BC prognosis (10, 18). However, the role that host immunity plays in shaping clinical outcomes requires further clarification through more comprehensive analyses.

To date, ICB therapy has been used successfully to treat patients with melanoma and other cancers (19–22), with three US Food and Drug Administration (FDA)-approved therapeutic vaccines used currently in clinical settings (23). However, fewer than 15% of cancer patients respond to ICB, although increased survival of some patients with solid tumors has been reported after immunotherapeutic treatment (24). Meanwhile, clinical studies of ICB and vaccine therapies have shown no significant immune modulation-related effect on TNBC treatment outcomes or patient survival (25, 26), while promising results have been obtained in clinical trials of immunomodulators administered with other treatments. For example, results of one clinical study demonstrated that administration of anti-programmed cell death protein 1 (PD-1) monoclonal antibodies prior to tumor resection enhanced local and systemic antitumor immune responses (27). In addition, results of a phase II study of glioma vaccines administered with granulocyte-macrophage colony-stimulating factor, cyclophosphamide and bevacizumab showed improved patient survival (28). Moreover, results of related studies have shown that during tumorigenesis, cytokines increase cellular oncogenic potential by promoting epithelial-mesenchymal transitioning, angiogenesis, immunosuppression, metastatic niche development and therapeutic drug resistance, as well as widespread TME changes and activation of intracellular signaling pathways. Therefore, oncologic cytokine studies may provide important insights into tumor immunology and reveal potential applications for regulatory cytokine-chemokine therapies in cancer treatment (29).

The TNBC-associated TME, which is highly complex and heterogeneous, exerts unclear effects on TNBC immunotherapeutic efficacy. In this study, TME characteristics were identified using nonnegative matrix factorization (NMF)-based virtual microdissection analysis, which can rapidly deconvolute gene expression data from tumor cells, inflammatory cells, stromal cells and cytokines in large numbers of tumor samples (30). Using this strategy, we extracted transcriptomic signals associated with expression of immunosuppression-related genes within the TME by analyzing RNA sequencing (RNA-seq) data from 303 human TNBC samples. Results of this analysis were then used to identify and validate TNBC TME immune cell types with immunosuppressive molecular signatures that potentially contribute to ICB resistance. These results were then used to formulate patient prognosis models and clinical treatment plans based on multiple variables.

## Materials and methods

### Data download and processing

A total of three publicly available datasets derived from The Cancer Genome Atlas (TCGA), Molecular Taxonomy of Breast Cancer International Consortium (METABRIC) and Gene Expression Omnibus (GEO) databases served as sources of data for study cohorts designated A, B and C, respectively. A survival prognosis model was developed based on the 303 TNBC patients of cohort A, of whom some patients were assigned to the early-stage group (I~II, 83 patients) for training and the remaining patients were assigned to the late-stage group (IIA~IV, 220 patients) for internal validation (31). To validate the established model, cohort B gene expression data (derived from METABRIC) derived from 154 paracancerous samples and 209 BC samples were employed (32). Thereafter, the survival prognosis model was validated for different TNBC subtypes by performing virtual microdissection analysis of cohort C data (accession number GSE16446) (33, 34) followed by survival analysis performed using Kaplan-Meier estimation analysis.

### Identification of the immune depletion class using unsupervised clustering analysis

**Figure 1:**
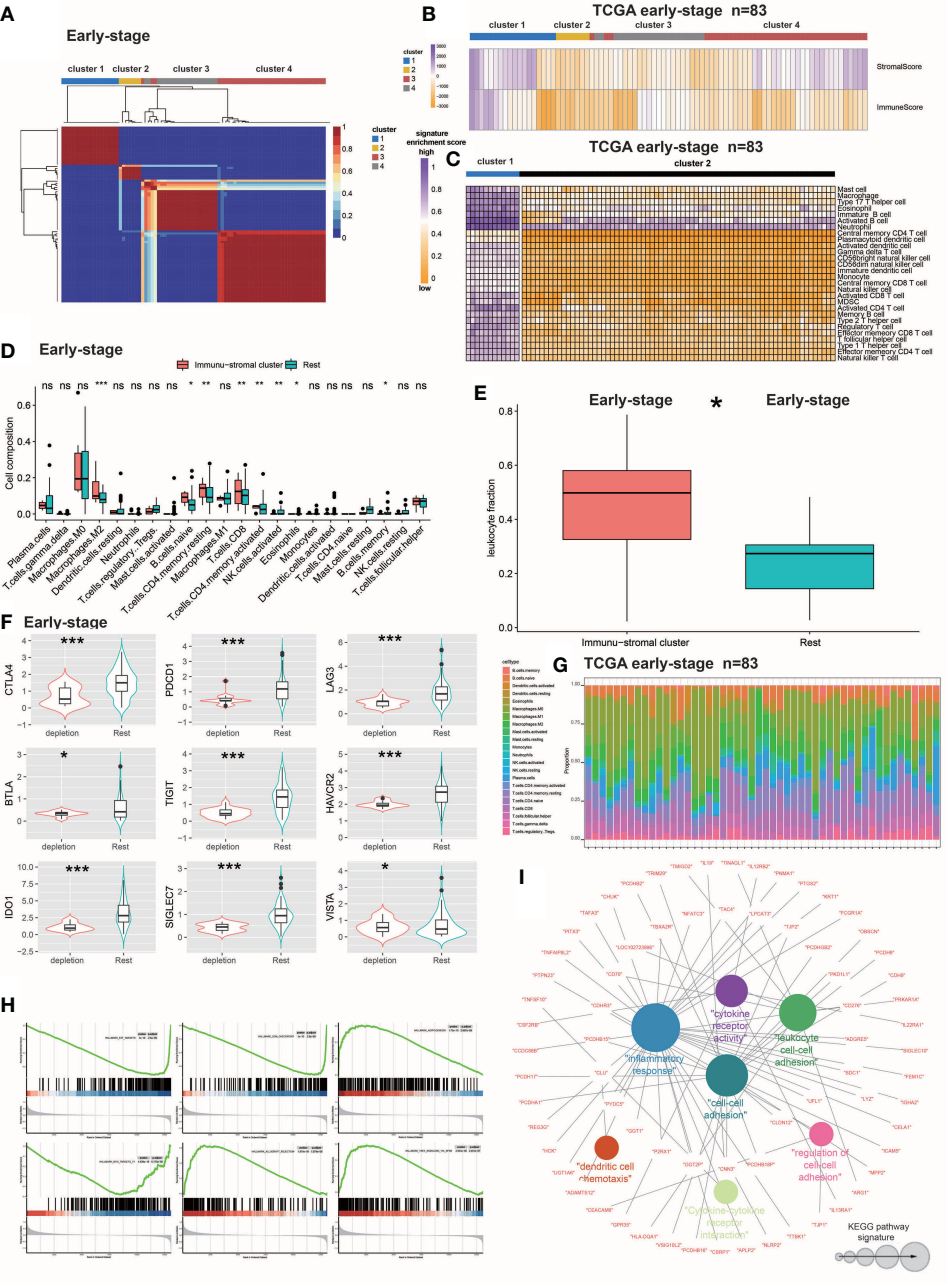
Identification and molecular characterization of IDC. **(A)** Heatmap of gene expression clusters and distinct expression patterns of 691 early-stage (III) TNBC samples from unsupervised NMF. **(B)** Matrix and immune enrichment scores for clusters of four expression patterns. High and low gene enrichment scores are depicted in purple and orange, respectively. **(C)** Enrichment fractions of gene signatures identify immune stroma and other clusters of immune cells. **(D)** CIBERSORT-inferred absolute fraction of TME cells compared between the two classes. **(E)** Box plot showing the difference in Leukocyte fraction between the two classes. **(F)** Box plots showing differences in the expression levels of various inhibitory receptors in rest clusters and in the immune and stromal cluster. **(G)** Histogram of the percentage of immune cells in each sample. **(H)** GSEA analysis reveals that IDC shows significant enrichment of marker gene sets related to immune cell metabolic processes. **(I)** KEGG pathway functional grouping network by ClueGO/CluePedia. Colorless and colored nodes represent metagene-specific genes and KEGG pathway terms, respectively. Node colors represent different functional groups. Node size represents the importance of KEGG pathways. The more important the KEGG pathway, the larger the highlighted node. All statistical differences between the two categories were compared using the Wilcoxon rank sum test. ns, >0.05; *P< 0.05; 0.01; ***P< 0.001.

First, bulk RNA-seq-based gene expression profiles of the 691-patient training cohort were subjected to virtual microanatomical analysis using NMF in R using one of the most critical NMF parameters, the decomposition level r, to define the number of clusters. When r was set to 4, the highest co-occurrence correlation coefficient was attained and the TCGA training cohort dataset was efficiently deconstructed (**Figure 1A**). Based on this result, r was set to 4. Following the method used in a recently reported study, immune and stromal enrichment scores were determined using single-sample gene set enrichment analysis (ssGSEA) (35, 36), which was bundled in GSVA to enable the identification of immune-related and stromal-related expression profiles (15). Once the immune and stromal cell enrichment scores were combined with the four NMF-identified clusters (numbered 1-4), we noticed that cluster 1 yielded stronger enrichment scores than the other clusters. As a result, cluster 1 was considered to be the “immune stromal cluster” within this context (37).

Second, numbers of certain types of immune cells present within immune stromal cluster tumors were studied by collecting data signals representing various immune cell subtypes while enrichment scores were determined according to ssGSEA-derived expression profiles. To evaluate the number of individual immune cells within the immune stromal clusters, enrichment fractions of 28 immune cells were merged with the clusters. Next, absolute fraction data of 22 infiltrating immune cells that were predicted by the CIBERSORT algorithm (according to gene expression patterns) were collected from the TIMER database (http://timer.cistrome.org/infltration estimation for tcga.csv.gz). Thereafter, leukocyte fraction data (TCGA all leuk estimate.masked.20170107.tsv) obtained from Thorsson et al. (https://gdc.cancer.gov/about-data/publications/panimmune) were used to determine DNA methylation-based signatures (38). Leukocyte fraction data were then obtained here based on analysis of images of TCGA tumors (including TNBC tumors) that can be found in the Supplementary Table (**Table S1**) of the Saltz study (39). To confirm lymphocyte enrichment within the immune stromal clusters, the immune-stromal class data were compared to that of the remaining clusters.

Finally, multiple inhibitory receptor expression profiles were analyzed that revealed immune stromal clusters that overexpressed multiple inhibitory receptors, of which a large proportion of signatures associated with T cell depletion were found to be enriched. As a result, the patients within the immune stromal cluster was designated as the IDC, while the remaining population was designated as the resting class.

### Validation of the late-stage IDC to confirm immune cell depletion

To confirm the presence of an immune-depleted state in advanced TNBC patients, the abovementioned methodologies for evaluating RNA-seq-based bulk gene expression profiles of the 619 early-stage TNBC samples were used to analyze corresponding profiles for the 210-sample late-stage cohort. Ultimately, four clusters were identified for the late-stage TNBC cohort (as for the early-stage TNBC cohort). Of these, cluster 2 yielded higher immune cell, stroma and TEX-related signature enrichment scores after signature enrichment scores from late-stage samples of the early TNBC cohort were incorporated while calculating the late-stage cohort signature enrichment score. As a result, cluster 2 was selected to be the IDC of the advanced TNBC group. After proportions of immunological cells and white blood cells and expression levels of various inhibitory receptors were compared between IDC and resting groups, gene set enrichment analysis (GSEA) was conducted to assess enrichment levels of markers and pathways then IDC scores were calculated based on ssGSEA of enrichment scores of 157 deficient immune function-related genes obtained during the training phase. The predictive potential of IDC scores was assessed using receiver operating characteristic (ROC) analysis.

### Correlations between IDC and resting class PD-L1 and TGF-β expression and the ICB response

Patient ICB treatment responses were predicted using the Tumor Immune Dysfunction and Exclusion (TIDE) algorithm. To investigate IDC patient responses to ICB treatment, programmed death-ligand 1 (PD-L1) expression was compared between IDC and resting classes. Higher TIDE prediction scores were generally associated with worse ICB responses.

According to Mariathasan et al., the cytokine transforming growth factor-β (TGF-β encoded by TGFB1) inhibits antitumor immunotherapies (40). By contrast, therapeutic co-administration of anti-TGF-β blockade-inducing antibody and anti-PD-L1 antibody reduced TGF-β signaling in stromal cells, promoted T cell infiltration into the tumor center and stimulated strong antitumor immunity and tumor regression. Taken together these observations suggest that TGF-β acts by limiting T cell infiltration of the TME to thereby suppress antitumor immunity. Therefore, detection of TGF-β in TNBC patient tumors correlates with resistance to antitumor immunotherapies.

### Construction of clinically relevant prognostic models

The R language LIMMA package was used to identify differentially expressed genes between immune cell-deficient TNBC and other TNBC samples and univariate COX regression analysis was performed (41). Thereafter, least absolute shrinkage and selection operator (LASSO) regression analysis was conducted to identify significant genes associated with survival for use in building a risk prediction model. The formula for calculating the risk score was as follows:


(1) $$\text{Risk score}=\;\sum_{\text{i}=1}^{\text{n}}\text{coefi X id}$$

where coefi is the coefficient and X is the normalized count for each gene. Based on the median risk score, we assigned patients to high-risk and low-risk survival-based groups. To test the model, follow-up TNBC data obtained from TCGA and metabric databases were used as training and test datasets, respectively. The reliability of the risk scoring model was assessed by within-group validation based on survival curves plotted based on training and test datasets then 1-, 3- and 5-year survival rates of patients were predicted based on the model. Thereafter, risk scores and clinical characteristics were evaluated together to obtain clinical information related to TNBC patient survival and prognosis followed by the creation of a forest plot of clinical prognostic factors.

### Correlation of drug sensitivity with TNBC gene expression profile

Drug response and drug-targeting pathway information were obtained by searching the Cancer Drug Sensitivity Genomics (GDSC) website (https://www.cancerrxgene.org/). Next, sensitivity data of two TNBC cell lines to different drugs were obtained then the pRRophetic package in R language was used to predict drug sensitivities of different TNBC cell phenotypes as based on gene expression data (42). These results were then used to generate risk scores for different drugs as a basis for clinical drug selection.

### Reverse transcriptase-PCR Analysis

Human breast cancer paracancerous cell line (MCF-10A) and human triple-negative breast cancer cell line (MDA-MB-453, MDA-MB-231) were purchased from Shangcheng North Na Chuanglian Biotechnology Co., LTD. MCF-10A was combined with MDA-MB-453 and MDA-MB-231 respectively constructed control groups to analyze the expression differences of PDCD1 and TGFB1 in different triple-negative breast cancer cell lines and normal breast cell lines. Total RNA was extracted using the Redzol kit from Beijing SBS Gene Technology Co., Ltd., and was extracted according to the instructions.

The forward primer sequence is TGFB1: F-5’-TTGACTTCCGCAAGGACCTC-3’, the reverse primer sequence is TGFB1: R-5’-ATCCGCAGTCCTCTCTCCAT-3’, and the product length is 421bp; PDCD1: F-5’-TGACTTCCACATGAGCGTGG-3’, the reverse primer sequence is PDCD1: R-5’-GCTCCTATTGTCCCTCGTGC-3’, and the product length is 294bp. qRT-PCR was performed using the SYBR^®^ Premix Ex Taq™ II (Takara, Shiga, Japan) Kit and a StepOnePlus Real-Time PCR instrument. Briefly, the mixture contained SureScript RTase Mix (20×) 1 μL, SureScript RT Reaction Buffer (5×) 4.0 μL, Total RNA 1 μg and dd HO (RNase/DNase free) supplemented to 20 μL, then sealed the cDNA with a transparent sealing film Array, mix well, spin off for 5s, 5 min at 25°C, 15 min at 42°C, 5 min at 85°C, keep at 4°C, and store the product at -20°C. qRT-PCR reactions were performed on a LightCycler 96 Real-Time PCR System (Roche Diagnostics, Indianapolis, IN). The reaction mixture was activated at 50°C for 2 min, pre-denatured at 95°C for 10 min, and then subjected to 40 cycles of amplification reactions at 95°C for 15 s and 60°C for 30 s. Finally, LightCycler 96 software (version 1.1.0.1320, Roche) was used for the collection and analysis of qRT-PCR data. With β-actin as an internal reference gene, the relative expression of mRNA was calculated by 2^-ΔΔCt^ method.

### Statistical analysis

R (version 4.2.1, http://www.rproject.org) was used for statistical discrete analysis. The Wilcoxon rank sum test for continuous data was used to correlate IDC and the remaining categories with tumor-infiltrating lymphocyte (TIL) percentage, mutation number and neoantigen number. Overall survival (OS) data were analyzed using Kaplan-Meier estimates and log-rank testing. To discover variable combinations, we included all clinicopathological factors in the Cox model. P-values of ≤0.05 were considered statistically significant. Pearson’s correlation was used to assess the strengths of two-variable linear relationships. Maftools, which enables the visualization and analysis of somatic mutations and calculation of total somatic mutation numbers (43), was used to determine differences in numbers of mutations between the IDC and other classes. Genomic mutation data of all 303 TNBC tumor samples were obtained from the TCGA database.

## Results

### Identification and characterization of a novel TNBC IDC immunosuppressive signature

NMF analysis was performed on the large number of RNA-seq-based gene expression profiles that were obtained for 691 TNBC samples in the training cohort then TEX-associated TME transcriptome signals were extracted. The training cohort dataset was clearly divisible into four patient clusters (**Figure 1A**). TNBC patients in cluster 1 had high immune and stromal enrichment fractions as assessed by ssGSEA and batch RNA-seq-based gene expression profiling, indicating considerable enrichment of immune cell and stromal feature gene expression signatures. This cluster, hereafter referred to as the immunological stromal cluster (**Figures 1B, C**), was subsequently found to possess a high density of immune cell signatures (**Figure 1D**) associated with immune cell subsets such as M2 macrophages, B cells, CD8^+^ T cells and eosinophils. Furthermore, the immune stromal cluster possessed a considerably greater proportion of leukocytes, as estimated from DNA methylation data, than did the other clusters (**Figure 1E**). Numbers of TILs detected during pathological examinations of images of tumors were also considerably higher in the immune stromal class than in the other clusters (**Figure 1F**). After the enrichment of immune cells in the immune stromal class was confirmed, absolute proportions of immune cells in the immunological stromal class and remaining clusters were compared using CIBERSORT analysis of batch RNA-seq data (**Figure 1G**).

To investigate TEX signaling function in TNBC, expression levels of several inhibitory receptors of immune system receptors, including CTLA-4, PDCD1 (also known as PD-1), BTLA, LAG3, TIGIT, HAVCR2 (also known as TIM-3), SIGLEC7, IDO1 and VISTA, were measured. Based on results of inhibitory receptor expression analyzes and enrichment scores for the TEX signaling gene set, a novel IDC subpopulation of the immune stromal cluster was identified that accounted for 21.4% of the population of the training cohort (**Figure 1H**). The resting class was defined as the resting subpopulation of the training cohort.

To define IDC-associated molecular markers, GSEA based on gene expression profiles of the training cohort was conducted that led to the identification of six marker gene sets that were enriched in the IDC molecular signature, with especially high enrichment noted for immunocytokine-related pathways and markers. Previously reported research results indicated that exhausted T cells can undergo apoptosis, as consistent with results obtained here showing that apoptotic markers were considerably elevated in the IDC of the early-stage TNBC cohort as evidence of severe T cell depletion. Results obtained using NMF analysis of IDC gene expression patterns led to the identification of 273 metagenes (sets of genes with coordinated expression) that after ClueGO network analysis were grouped into Kyoto Encyclopedia of Genes and Genomes (KEGG) pathway networks that revealed their biological functions (**Figure 1I**). The majority of genes within these metagenes were functionally linked to cytokines and associated molecules that mediate immunological and inflammatory responses. These 76 immune function-related genes, which included members of the immunoglobulin superfamily (CDH9 and CDHR3), interleukin receptor molecules (IL9, IL19, IL13RA1 and IL12RB2) and cytokines (CD70 and CD276) were then utilized to validate TNBC patient immunological exhaustion (**Supplementary Tables S2**, **S3**). Taken together, these results demonstrated that we effectively identified an IDC gene expression pattern indicating severe T cell depletion within the TME of some TBNC patients.

### Internal validation of the late-stage TCGA TNBC IDC immunosuppressive molecular signature

**Figure 2:**
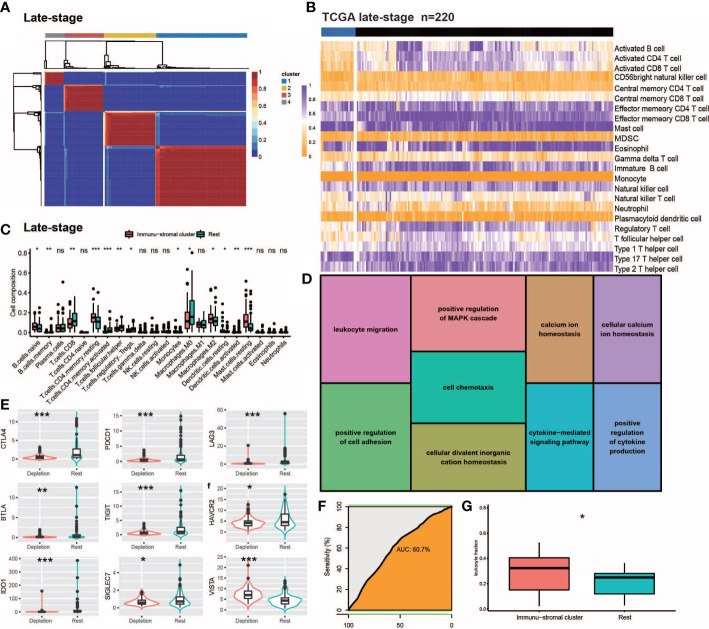
Internal validation of IDC in late stage TCGA-TNBC samples. **(A, B)** Consensus clustering for the late stage TCGA-TNBC. **(C)** The comparison of the absolute fractions of TME cells inferred by CIBERSORT between two classes. **(D)** Kegg pathway enrichment visualization in immune depletion Classifer Genes. **(E)** Boxplots shows the different expression levels of multiple inhibitory receptors between two classes. **(F)** ROC curve evaluated the predictive capacity of 157 immune depletion classifer genes in late stage TCGA-TNBC samples. **(G)** Box plots show the differences of leukocyte fraction between two classes. All statistical differences of two groups were computed by Wilcoxon rank-sum test; *P< 0.05; **P< 0.01; ***P< 0.001.

We conducted NMF analysis of bulk RNA-seq-based gene expression profiles of TCGA late-stage samples to confirm the presence of reduced immune classes in the TNBC TME, with results of NMA leading to identification of four clusters (**Figure 2A**). When these clusters were combined with ssGSEA-calculated feature enrichment scores and bulk RNA-seq-based gene expression profiles, cluster 1 was found to possess higher stromal and immune enrichment scores than the other clusters (**Figure 2D**). Along with other TEX-related cytokine indicators, these features were considerably enriched (**Figures 2B, C**). As a result, cluster 1 was designated as the IDC of the TGCA samples, as consistent with observed co-upregulation within this cluster of numerous inhibitory receptors (**Figure 2E**) that were enriched for Gene Ontology (GO) functional terms leukocyte migration, positive regulation of the MAPK cascade, cell chemotaxis, positive regulation of cell adhesion, cellular divalent inorganic cation homeostasis, positive regulation of cell adhesion, calcium ion homeostasis, cytokine-mediated signaling pathway, cellular calcium ion homeostasis and positive regulation of cytokine production (**Figures 2D, E**). Notably, ROC curve-based results (**Figure 2F**) confirmed that the 167 depleted immune function-related genes may be useful for predicting the presence of IDC immunosuppressive signatures in TMEs of individual TNBC patients.

### Poor prognosis of TNBC patients with immunosuppressive IDC signatures

**Figure 3:**
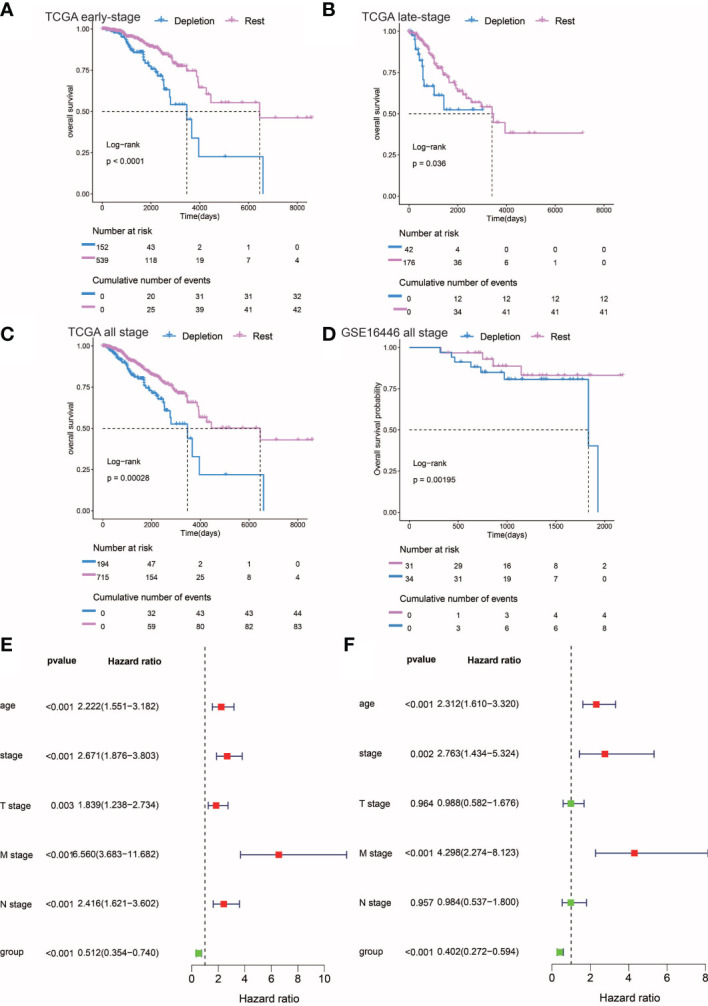
Prognostic analysis of different stages of IDC and rest types of TNBC. **(A, C)** Kaplan-Meier estimates of overall survival for IDC and rest classes of advanced, early, and persistent TNBC. **(D)** Kaplan-Meier estimates of overall survival for IDC and rest classes of TNBC in the gse16446 dataset. P-values were calculated using the log-rank test. **(E, F)** Multivariate and univariate Cox regression analysis (group, tumor stage, and age) for full-stage TNBC.

In order to explore the utility of reduced immune function status for predicting TNBC patient prognosis, we correlated categories with clinicopathological factors. Previous studies had linked high-density TILs with better outcomes, such as improved overall survival (OS) (44, 45). Here, IDCs of both early and advanced TNBC patients possessed higher proportions of TILs than did IDCs of other patient categories. However, Kaplan-Meier estimates of early and advanced TNBC OS rates revealed significantly lower OS rates in patients with TME IDC immunosuppressive signatures than in TMEs of the resting group (P< 0.0001; **Figures 3A, B**), while in both early and advanced TNBC, multivariate survival analysis conducted using Cox regression models showed that the IDC immunosuppressive signature was an independent predictor of OS (P< 0.001). (**Figures 3E, F**). Finally, the prognostic value of IDC immunosuppressive signatures in TCGA and GEO cohorts of TNBC patients in all disease stages were studied. As expected, IDC patients had worse OS than the resting groups (**Figures 3C, D**). Taken together, these findings suggest that although T cells are abundant in IDCs, most of these T cells have lost effector functions for limiting tumor growth, resulting in a state of immunosuppression that leads to continued tumor growth and poor prognosis. Survival data also confirmed that IDC signatures of patients with late-stage TNBC exhibited more pronounced T cell depletion than those of early-stage TNBC patients.

### The IDC immunosuppressive signature correlates with immunotherapy resistance

**Figure 4:**
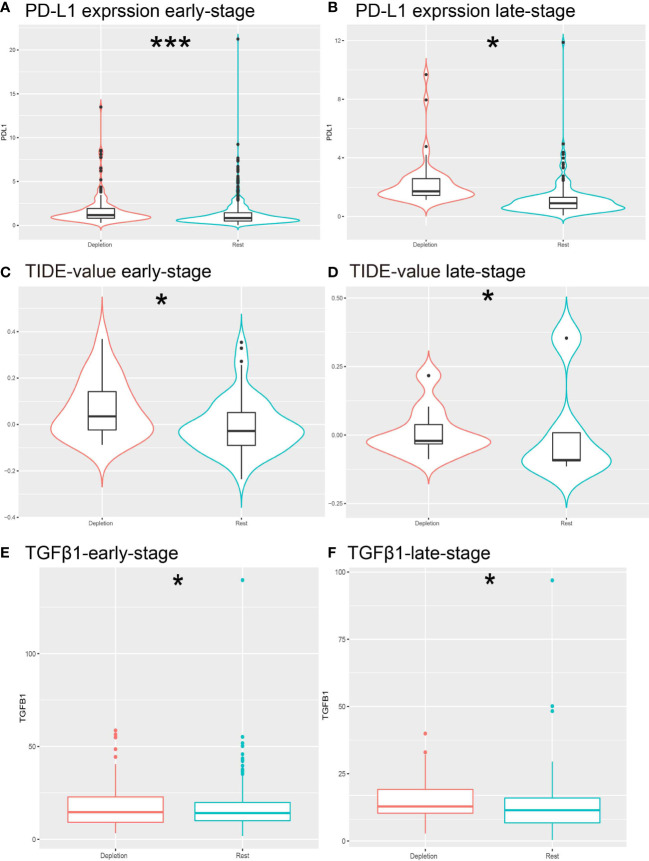
Prediction of resistance to ICB therapy. **(A, B)** Different expressions of PD-L1 expression levels in different stages of IDC patients. **(C, D)** Different expression of TIDE predictive score for each stage of ICB treatment. **(E, F)** Different expression of TGFb1 in IDC and rest classes.*: *P< 0.05; ***P< 0.001.

Treatment of cancer patients with therapeutic antibodies targeting the PD-L1 pathway can elicit long-lasting, robust responses. However, efficacies of such treatments are frequently reduced due to the emergence of drug resistance. To study the response of the IDC group to ICB treatment, we compared PD-L1 expression levels between IDC and resting patient groups and found that PD-L1 expression levels were higher in the early IDC group than in the resting group (**Figures 4A, B**). Next, use of the Tumor Immune Dysfunction and Rejection (TIDE) algorithm to predict ICB treatment responses revealed that both early and advanced TNBC IDCs had higher TIDE prediction scores than did the other categories (**Figures 4C, D**), such that a higher TIDE prediction score indicated a worse ICB response. Importantly, TGFβ1 was expressed at a lower level in resting classes than in IDCs (**Figures 4E, F**), which is consistent with results obtained by Mariathasan et al. that indicated the cytokine TGF-β (encoded by TGFβ1) suppresses effects of antitumor immunotherapy (46). Taken together, these results suggest that the IDC immunosuppressive signature has a ICB therapy resistance.

**Figure 5:**
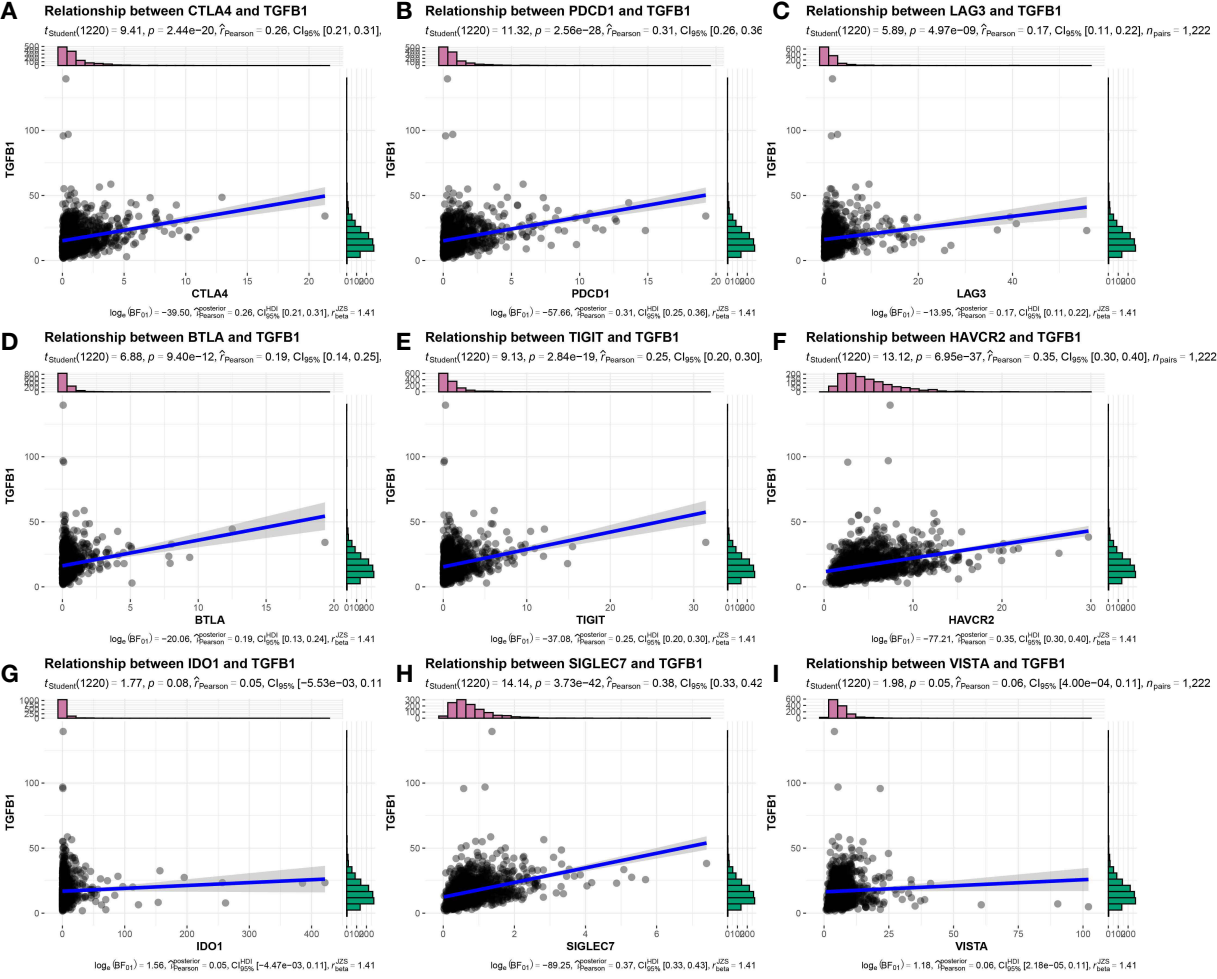
Expression correlation of TGFβ1 and immune checkpoint-related genes in TNBC patient samples. **(A)** CTLA4, **(B)** PDCD1, **(C)** LAG3, **(D)** BTLA, **(E)** TIGIT, **(F)** HAVCR2, **(G)** IDO1, **(H)** SIGLEC7, **(I)** VISTA.

Considering that tumors that escape immune editing in cancer patients often express molecules that suppress the antitumor immune response, such as PD-L1 (also known as B7-H1), indoleamine 2,3-dioxygenase (IDO) and others, here expression levels of these molecules were correlated with TGF-β expression using Pearson correlation analysis. As expected, expression levels of these molecules were positively correlated with TGFβ1 mRNA expression levels (**Figures 5A–I**), thus suggesting that IDC patients remain in an ICB-unresponsive state, despite higher PD-L1 expression levels and that anti-PD-L1 therapies. In addition, these results suggest that *in vivo* antitumor response molecules play key roles in this process.

### IDC molecular signatures possesses no significant tumor mutational differences

**Figure 6:**
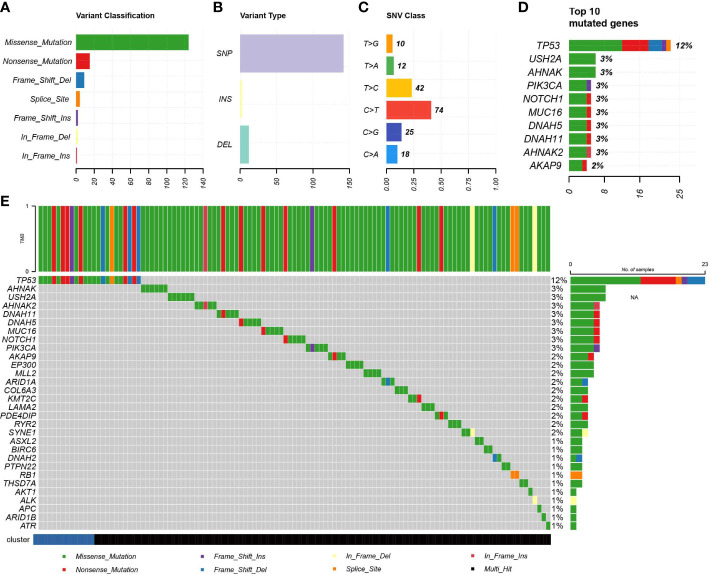
Association of IDC with somatic mutations. **(A, B)** Summary of mutation information for TNBC samples in the TCGA database. **(C) **Summary of variant Classification within TNBC. **(D) **Top 10 triple negative breast cancers mutated genes. **(E)** The landscape of most frequently mutated genes between the IDC and the rest class in TNBC.

Due to the fact that genomic mutations occurring in tumors are strongly associated with immunotherapy outcomes, we explored the landscape of commonly mutated genes between TNBC IDC and resting class molecular signatures. In the TCGA cohort, censorship mutations were the most common TNBC mutations detected (**Figure 6A**), of which most mutations were SNPs (**Figure 6B**) that most often contained C>T base substitutions (**Figure 6C**). The ten genes with the highest mutation frequencies were TP53, USH2A, AHNAK, PIK3CA, NOTCH1, MUC16, DNAH5, DNAH11, AHNAK2 and AKAP9 (**Figure 6D**), with no significant differences in individual gene mutations observed between IDC and resting group signatures (**Figure 6E**). These results thus suggest that somatic mutations are not significantly associated with immunosuppressive TME status.

### Construction and validation of the prognostic model

**Figure 7:**
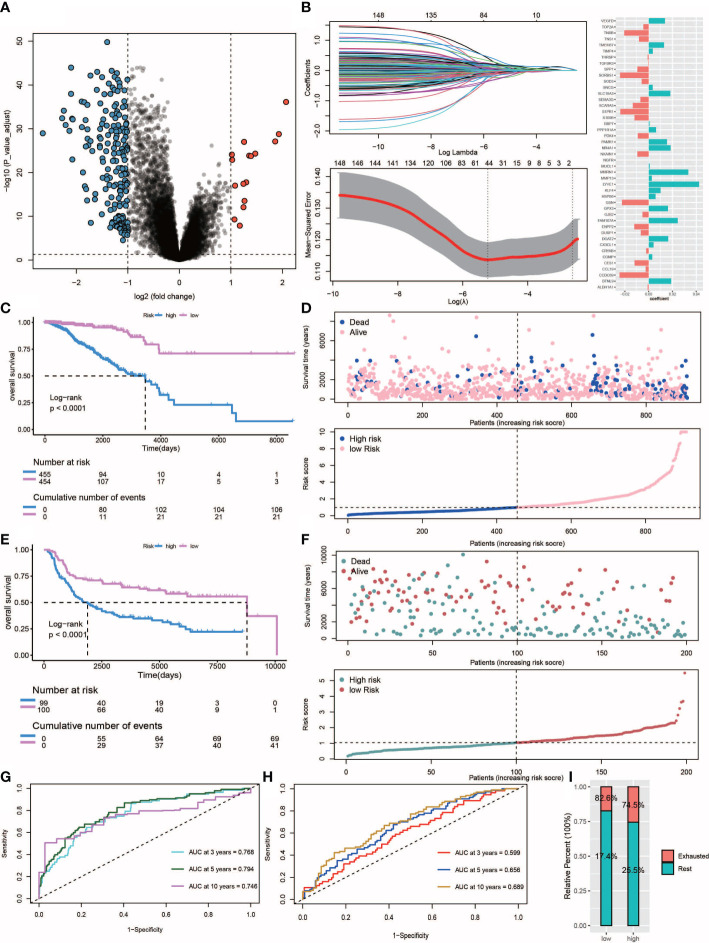
Construction and validation of the risk score. **(A)** Volcano plot of differentially expressed genes between IDC and rest class samples, blue indicates down-regulated expression and red indicates up-regulated expression. **(B)** Lasso regression analysis and multivariate stepwise Cox regression analysis for identification of the immune risk signature. **(C, E)** Kaplan-Meier curves of training set (p<.001, log-rank test) and test set (p<.001, survival rate comparison). **(D, F)** Association between patient survival and increased risk score. **(G, H)** Time-dependent receiver operating characteristic (ROC) of training and test sets. **(I)** Proportion of IDC and rest class in high and low risk patients.

Ultimately, a total of 211 differentially expressed genes associated with survival were identified (**Figure 7A**, **Supplementary Table S4**). The univariate Cox regression algorithm was used to initially obtain 150 genes related to TNBC patient prognosis then hazard ratios (HRs) and P values of these genes were calculated (**Supplementary Table S5**). Next, we constructed risk models using the LASSO algorithm that ultimately led to the identification of 45 prognostically relevant genes (**Figure 7B**). These genes were then used to construct risk score-based models based on training (n = 303) and test (n = 211) datasets obtained from TCGA-TNBC and metabric TNBC patients, respectively. Results of survival analysis revealed that higher risk scores in training and test sets corresponded to poorer survival (P< 0.0001) (**Figures 7C–F**). Time-dependent ROC curves were generated and used to assess the sensitivity of the prognostic model. Results obtained for the areas under ROC curves (AUCs) revealed 3-, 5- and 10-year AUCs for the training set of 0.768, 0.794 and 0.746, respectively (**Figure 7G**), and corresponding AUCs for the test set of 0.599, 0.656 and 0.689, respectively (**Figure 7H**). Additionally, multivariate Cox regression analysis was conducted to assess whether clinical characteristics, such as age, cancer stage, TNM stage and risk scores were independent factors related to TNBC patient prognosis (**Supplementary Figure S2**). We found that age, tumor stage and risk score were independent prognostic factors for both training and test sets of TNBC patients. Notably, more high-risk patient molecular signatures contained IDC signatures (**Figure 7I**), suggesting that immune system failure may be responsible for poor survival of high-risk patients.

### IDC immunosuppressive signatures can be useful for predicting the chemotherapeutic response

**Figure 8:**
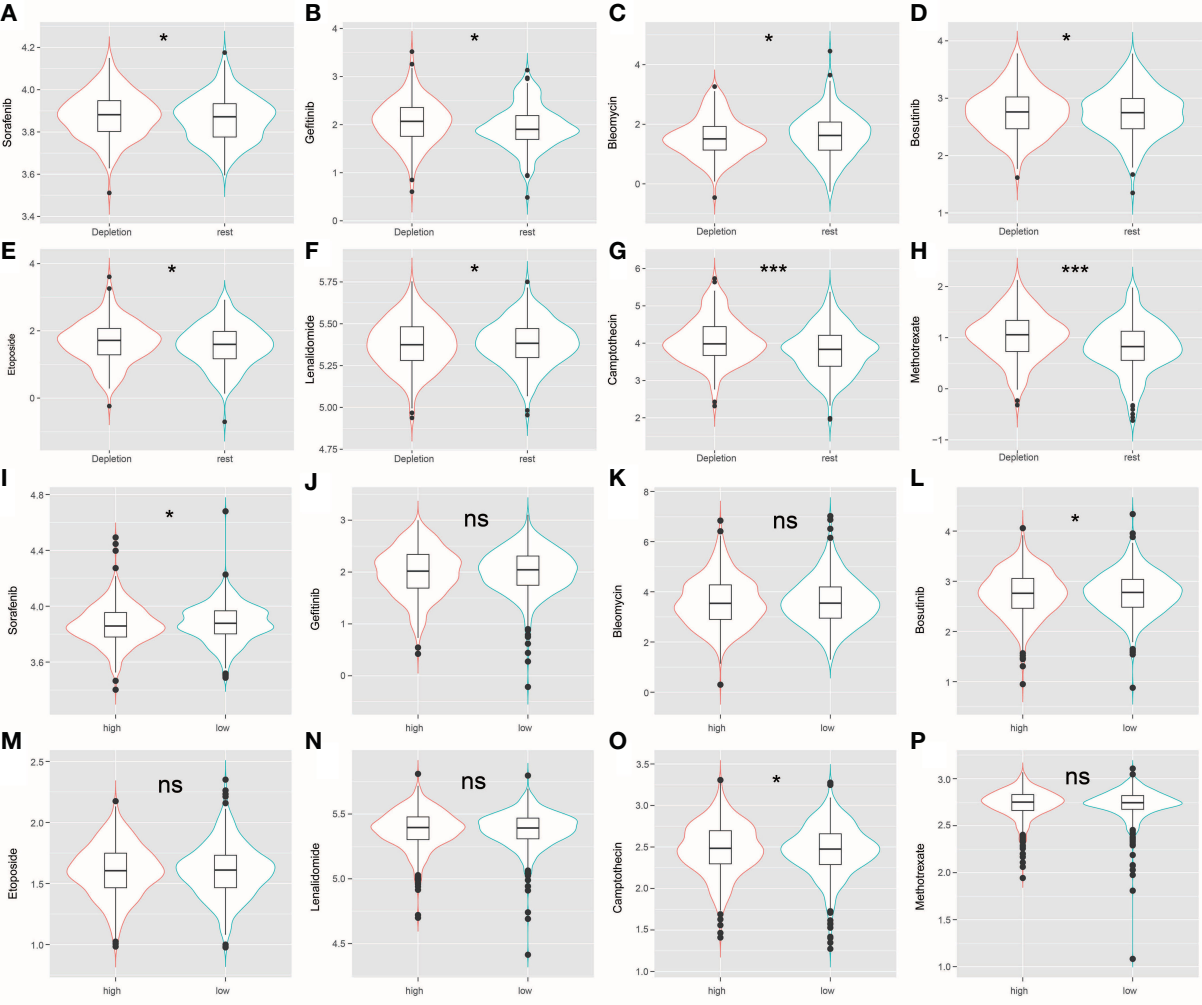
**(A–H)** The violin plot shows that drug sensitivity prediction score(Sorafenib, Gefitinib, Bleomycin, Bosutinib, Etoposide, Lenalidomide, Camptothecin, Methotrexate) are distributed differently among groups. **(I–P)** The violin plot shows that drug sensitivity prediction score(Sorafenib, Gefitinib, Bleomycin, Bosutinib, Etoposide, Lenalidomide, Camptothecin, Methotrexate) are distributed differently among risk groups; ns: no significance; *: P < 0.05; ***: P < 0.001.

The R package “pRRophetic” was used evaluate high-risk and low risk patient IDC, and resting class groups for chemotherapeutic responses and resistance. **Figure 8** shows drug sensitivity results for three types of triadic breast cancer cell lines to eight anti-cancer therapy drugs (sorafenib, gefitinib, bleomycin, bosutinib, etoposide, lenalidomide, camptothecin, methotrexate). Statistical analysis showed that, except bleomycin, the IC50 level of resting grade patients was higher than that of IDC patients. Sensitive (**Figures 8A–H**). In addition, for the risk group Sorafenib, the low-risk group had a higher score, while for Bosutinib and Camptothecin, the low-risk group had a lower score (**Figures 8I–P**).

### TNBC gene expression level verification *via* quantitative reverse transcription PCR

**Figure 9:**
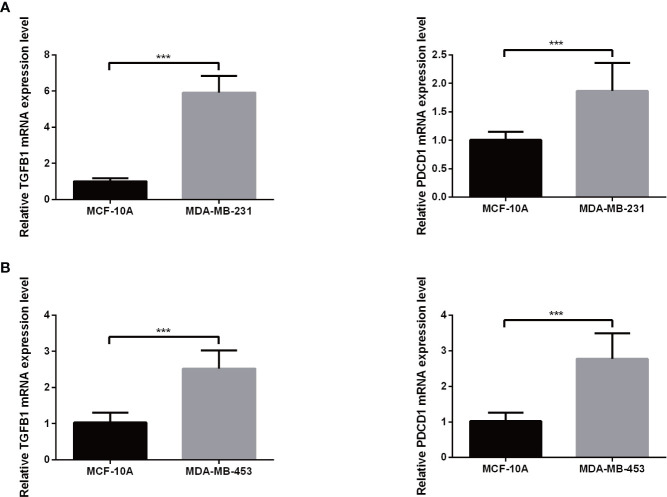
The mRNA expression of TGFB1 **(A)** and PDCD1 **(B)** in a TNBC cell lines and the adjacent cell lines. ***P< 0.001.

Expression levels of selected genes were determined using qRT-PCR to confirm their reliability. As shown in **Figures 9A, B**, the results for all mRNA transcripts showed that they were expressed at significantly higher levels in the two TNBC tumor cell lines (MDA-MB-231, MDA-MB-453) than in the adjacent normal cell lines.

## Discussion

The rise of immunotherapy in recent years has revolutionized TNBC treatment by significantly improving overall patient survival. Nevertheless, high PD-L1 expression levels detected in more than half of TNBC patients indicate that their tumors are resistant to immune checkpoint inhibitors (47), while side effects of these inhibitors are common. Although our understanding of mechanisms underlying ICB resistance is extremely limited, data obtained to date suggest that an immunosuppressive TME comprised of tumor cells, immune cells and other stromal components may be involved in ICB resistance (48). Consequently, identification of ICB-resistant TNBC patients and optimization of immunotherapeutic protocols will require characterization of immunosuppressive TMEs at the molecular level.

In this investigation, NMF was utilized to deconvolve gene expression patterns of TME TEX, immune cells and stromal elements as derived from TNBC datasets. Our results led to the successful discovery of a new immunosuppressive TNBC category comprising 21.4% of TNBC patients. In contrast to IDCs observed in head and neck squamous cell and liver cancers (49, 50), TNBC IDCs possessed greater immunological and stromal enrichment scores, indicating the presence of numerous immune cell and stromal components. As expected, IDC classes exhibited particular characteristics, such as significant immune cell infiltration, co-upregulation of several inhibitory receptors, increased expression of immunosuppressive cytokines and elevated PD-L1 expression. Among TME-infiltrating immune cells, tumor-associated M2 macrophages and T cells have been reported to act as immunosuppressive cells that play important roles in immune evasion and reduction of ICB therapeutic efficacy (51, 52). Meanwhile, IDCs have been found to be widely distributed across different tumor stages, but may differ in levels of T cell depletion between early (stage I-II) and advanced (stage III-IV) TNBC samples. In fact, results of a previous study showed that severely depleted T cells may undergo apoptosis (53), as consistent with results of this study showing that apoptotic marker gene sets were enriched in late-stage IDC but not in early-stage IDC. Thus, the late-stage TNBC IDC immunosuppressive signatures are associated with higher levels of T cell exhaustion as compared to those of the early-stage TNBC IDCs.

Results of a previous study suggested that significant tumor mutational and neoantigen burdens correlated with the ICB therapeutic response (54). Surprisingly, mutational burden did not correlate with IDC or substantial lymphocytic infiltration in our study. In addition, TNBC tumor genetic variations (mutation and neoantigen burdens) between IDC and resting classes were similar to corresponding burdens observed for other malignancies, such as head and neck squamous cell and hepatocellular carcinomas (49, 50), thus suggesting that tumor-intrinsic mutations may not contribute to the immunosuppressive TEM phenotype. Moreover, resilience of IDCs was successfully validated from various viewpoints, although this result must be validated in TNBC patients treated with ICB. Furthermore, our results underscore the fact that a greater understanding of molecular properties of immunosuppressive TMEs is essential before successful immunotherapeutic treatments can be developed to reverse TEX and treat TNBC. In summary, here we defined IDC immunosuppressive signatures that permitted us to construct prediction models that may improve predictions of TNBC patient survival and assist clinicians in selecting appropriate immunotherapeutic treatments for individual TNBC patients.

Mariathasan’s research results have shown that therapeutic antibodies that block PD-1 and PD-L1 pathways can induce robust and durable responses in patients with various cancers (40). However, efficacies of these treatments are reduced when a fibroblast TGF-β signaling signature is detected, which is especially common in patients with tumors in which CD8+ T cells are excluded from the tumor parenchyma but are found in fibroblast-rich, collagen-rich peritumoral stroma. For such patients, we found that administration of a combination therapy consisting of TGF-β blockade and anti-PD-L1 antibody reduced TGF-β signaling in stromal cells, promoted T cell infiltration into the tumor center and triggered robust antitumor immunity and tumor regression. Taken together, these results indicate that TGF-β shapes the TME by limiting T cell infiltration to suppress antitumor immunity, a conclusion that was further supported by additional results obtained here showing up-regulated co-expression of TGFB1 and PDCD1 in TNBC cell lines.

As final considerations, more clinical data on TNBC patient responses are needed to validate our findings, while further explorations of potential applications of IDC immunosuppressive signatures are needed toward improving selection of cancer immunotherapy regimens and improving efficacies of these treatments. Furthermore, research studies exploring effects of targeted treatments on patient outcomes in clinical practice are needed that should include more comprehensive characterization of IDCs and their effects on TNBC patient therapeutic outcomes.

## Conclusion

In conclusion, we identified an immunosuppressive class, accounting for approximately 21.4% of TNBC patients, that exhibited potential resistance to ICB therapy and the unique immunosuppressive molecular signature of TME. Our findings provide new insights into understanding the molecular mechanisms of resistance to ICB therapy and tailoring appropriate immunotherapy strategies for patients with different molecular signatures.

## Data availability statement

Publicly available datasets were analyzed in this study. This data can be found here: https://portal.gdc.cancer.gov/.

## Author contributions

RD designed the study, performed the major data analysis, and drafted the manuscript. WS provided funding source, designed, oversaw, and supervised the project and edited, reviewed, and finalized the paper. All the authors were involved in experimental studies. All authors contributed to the article and approved the submitted version.
