# Supplementary figures and images for: Identification of immunosuppressive signature subtypes and prognostic risk signatures in triple-negative breast cancer
Source: Front Oncol. 2023 Jun 12;13:1108472. doi: 10.3389/fonc.2023.1108472 (PMC10292819; doi:10.3389/fonc.2023.1108472)

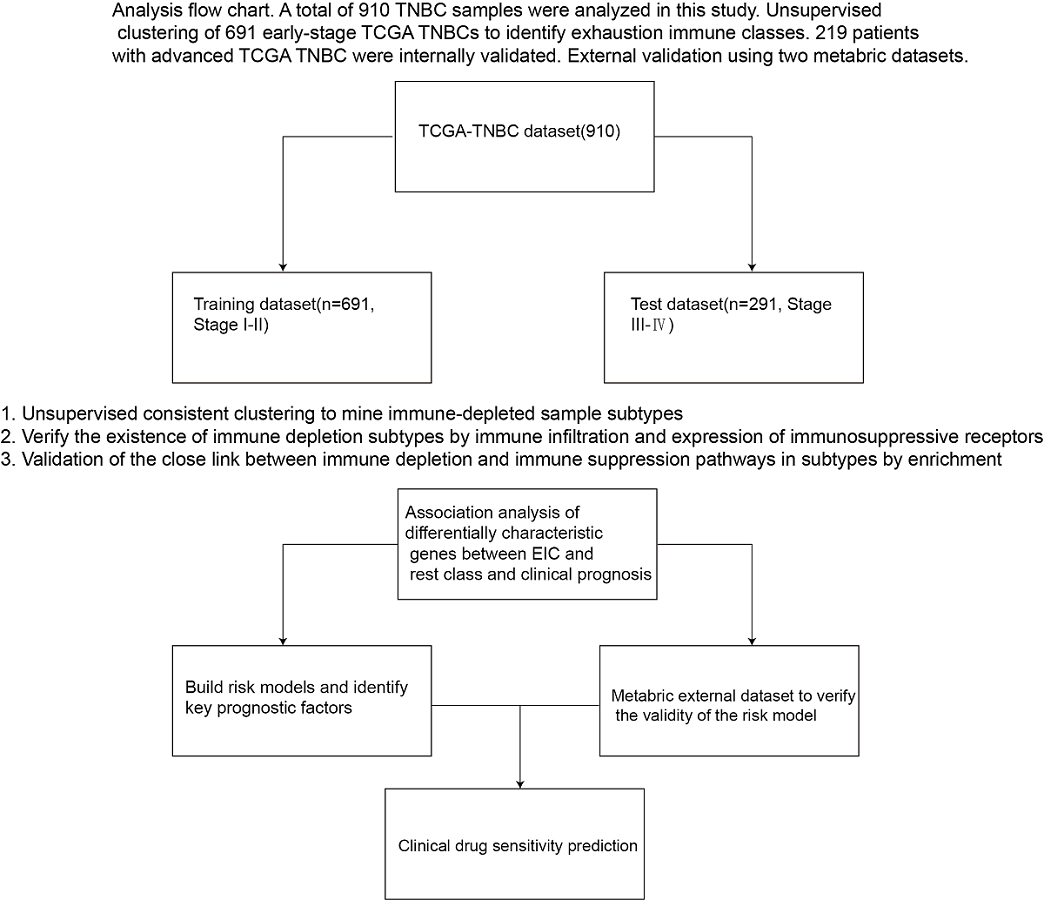

Supplement: Supplementary file 1 [file Image_1.tiff]

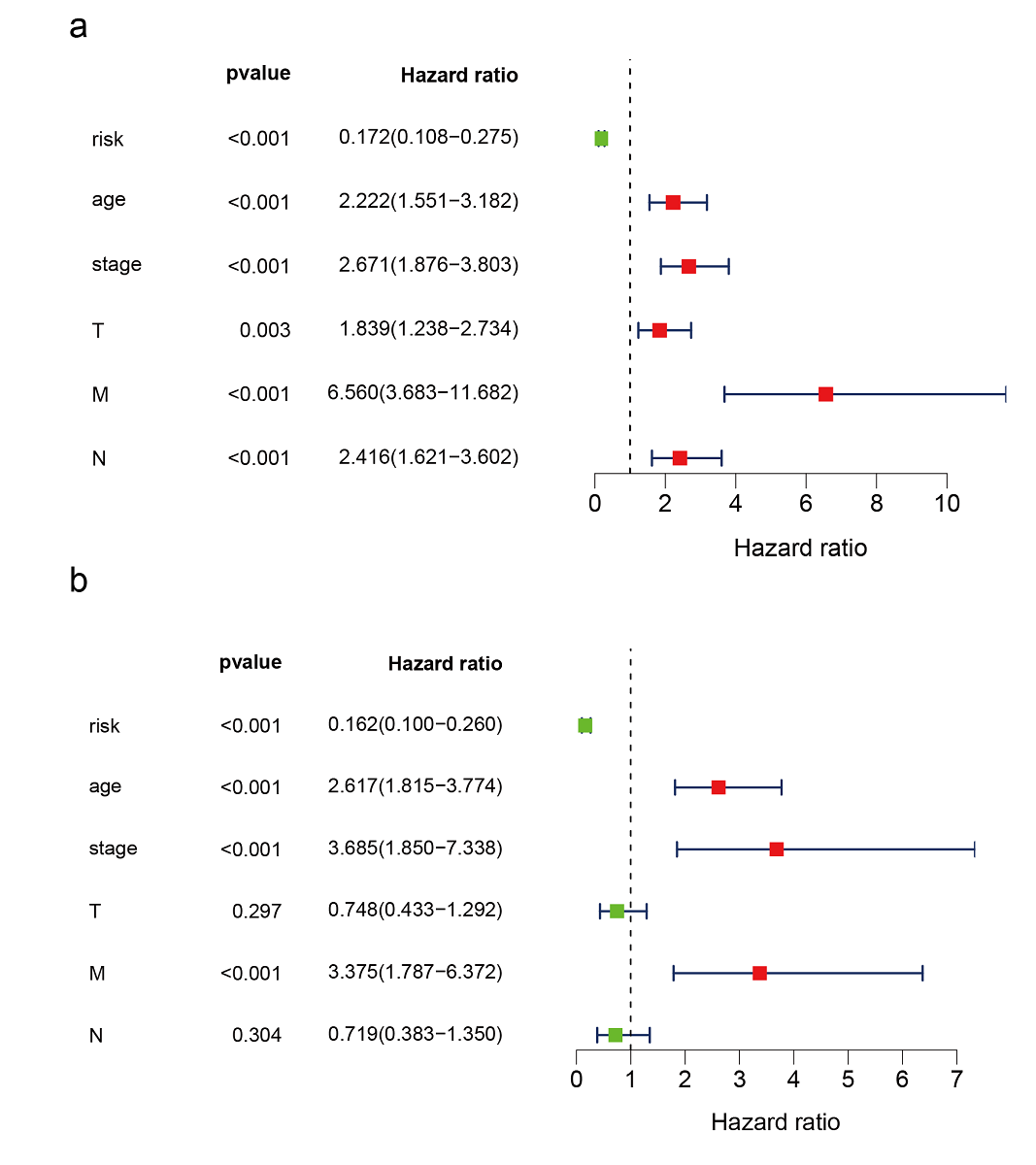

Supplement: Supplementary file 2 [file Image_2.tiff]
